# Soil respiration in a subtropical forest of southwestern China: Components, patterns and controls

**DOI:** 10.1371/journal.pone.0204341

**Published:** 2018-09-27

**Authors:** Kaijun Yang, Yulian Yang, Zhenfeng Xu, Qinggui Wu

**Affiliations:** 1 Ecological Security and Protection Key Laboratory of Sichuan Province, Mianyang Normal University, Mianyang, China; 2 Institute of Ecology and Forest, Sichuan Agricultural University, Chengdu, China; Tennessee State University, UNITED STATES

## Abstract

Partitioning the components of soil respiration is crucial to understand and model carbon cycling in forest ecosystems. In this study, total soil respiration (*R*_S_), autotrophic respiration (*R*_A_), heterotrophic respiration (*R*_H_), litter respiration (*R*_L_), litterfall input and environmental factors were synchronously monitored for 2 years in a subtropical *Michelia wilsonii* forest of southwestern China. *R*_H_ rates were often higher than *R*_A_ rates during the two years except for the middle growing season (from July to September). The mean rate of *R*s, *R*_A_, *R*_H_ and *R*_L_ was 1.94 μmol m^-1^ s^-1^, 0.85 μmol m^-1^ s^-1^, 1.09 μmol m^-1^ s^-1^ and 0.65 μmol m^-1^ s^-1^, respectively, during the 2-year experiment. Annual CO_2_ emission derived from *R*_A_, *R*_H_ and *R*_L_ was 3.26 Mg C ha^-1^ a^-1^, 4.67 Mg C ha^-1^ a^-1^ and 2.61 Mg C ha^-1^ a^-1^, respectively, which accounted for 41.4%, 58.6% and 32.9% of *R*_S_. Over the experimental period, the ratio of *R*_A_/*R*_S_ increased with soil temperature but the opposite was true for *R*_H_/*R*_S_ and *R*_L_/*R*_S_. The *Q*_10_ value was 2.01, 4.01, 1.34 and 1.30, respectively, for *R*_S_, *R*_A_, *R*_H_ and *R*_L_. Path analysis indicated that environmental variables and litterfall production together explained 82.0%, 86.8%, 42.9% and 34.7% variations of monthly fluxes of *R*_S_, *R*_A_, *R*_H_ and *R*_L_, respectively. Taken together, our results highlight the differential responses of the components of *R*_S_ to environmental variables.

## Introduction

Soil respiration (*R*_S_) is the second largest flux of carbon dioxide (CO_2_) between terrestrial ecosystems and the atmosphere [1, 2]. *R*_S_ accounts for roughly 80% of ecosystem respiration across global forests [3]. In general, *R*_S_ is largely controlled by environmental factors, including temperature and moisture [4]. Moreover, *R*_S_ is complicated by tree growth and the subsequent input of plant litter to soil [5, 6].

*R*_S_ is overwhelmingly the product of respiration by plant roots (autotrophic respiration, *R*_A_) and soil organisms (heterotrophic respiration, *R*_H_) [7, 8]. In addition, CO_2_ flux derived from decaying litter accounts for a considerable part of *R*_S_, which is strongly controlled by the quantity and quality of litter and climate variables [5, 8, 9]. A recent meta-analysis indicated that aboveground litter removal and root removal declined *R*_S_ by 22.8% and 34.1%, respectively [6]. Soil temperature is one of the most important factors that control the variations of *R*_S_ in terrestrial ecosystems but the size of this effect is dependent on system types and climate zones [10, 11]. Soil moisture also mediates the temporal and spatial pattern of *R*_S_ [4, 12]. Both *R*_A_ and *R*_H_ generally increased with temperature and precipitation across global forest ecosystems [4]. However, moisture effect may be complicated by the effects of soil temperature and other factors [13, 14]. Different components of *R*_S_ could be dominantly mediated by different mechanisms, such as substrate quality, plant traits and environmental factors. Obviously, partitioning the components of *R*_S_ and exploring the relative importance of biotic and abiotic factors on each component is very helpful for understanding the mechanistic of soil carbon cycling.

Chinese subtropical forests have high biomass and productivity, which play an important role in the carbon storage of global terrestrial ecosystem [15]. Over last decades, almost all natural forests in subtropical China have been deforested due to demand for timber, and subsequently often reforested with fast-growing tree species. So far, previous studies focused mainly on the plantations dorminated by non-native fast-growing tree species, such as *Pinus massoniana* and *Cunninghamia lanceolata* [16, 17]. However, the components of *R*_S_ and its controls have been scarcely investigated in the restored forests dorminated by native tree species in this region. In this study, we investigate the components of *R*_S_ for two years using root trenching and litter exclusion techniques in a subtropical *Michelia wilsonii* (a special native tree species) forest of southwestern China. The specific objectives of this study were (1) to explore the seasonal dynamics of each respiration component; (2) to assess the relative importance of biotic and abiotic factors for temporal patterns of each component.

## Materials and methods

### Ethics statement

We received a permission from the Dujiangyan Bureau of Forestry to conduct this experiment in the studied forest in 2015. In this study, only limited soil samples were collected to measure physical and chemical properties and PVC chambers were set up to monitor soil respiration. Our work had negligible effects on the function of the broader ecosystem. Additionally, this study was carried out in compliance with the laws of the People’s Republic of China. This study did not involve measurements of humans or animals, and no endangered or protected plant species were involved.

### Site description

The site is conducted in the Dujiangyan Experimental Forest of Sichuan Agricultural University, southwestern China (103° 37’ E, 30° 59’ N, 911 m asl). This area is characterized by a mid-subtropical, humid, mountainous climate that produces the wet season from May through October and the dry season from November through April. The mean annual temperature and precipitation is 15.2°C and 1 243 mm, respectively. The dominant tree species is *Michelia wilsonii* and the understory are *Lespedeza bicolor*, *Pittosporum glabratum*, *Dranceopteis dichotome* and *Cyperus rotundus*, respectively. The soil is classified as ferralsol with old alluvial yellow loam according to the Chinese Soil Taxonomy (RGCST 2001). The basic topsoil properties (0–20 cm) as determined in August 2016 are as follows: organic C 15.76 g kg^−1^, total N 1.92 g kg^−1^, total P 0.32 g kg^−1^, and pH 5.73. Neither fertilization nor drainage had been carried out since tree establishment in the stand. Moreover, the topography is relative flat (less than slope 10°) and tree canopy coverage is about 0.9.

### Experimental design

In August 2015, five 10 m × 10 m replicate plots were established in the experimental site. There were three treatments: control [C] (undisturbed), No-Roots [NR] (root growth excluded) and No-Litter [NL] (aboveground litter excluded). In each plot, a trench subplot was set up with a dimension of 1 m × 1 m. For the trenched subplots, we dug a trench of 0.2 m width and 0.6 in depth. The polyethylene films (37 μm mesh size) were placed along the bottom and sides of the trenches to prevent roots from entering the trench. The excavated soil was gently backfilled into the trench according to its initial profiles to minimize disturbance. Existing litters in the NL plots (1 m × 1 m) were removed and litterfall was excluded by pyramid-shaped screens (1-mm mesh) placed approximately 1 m aboveground. All litters fell around the NL plots were monthly removed before the measurements of soil respiration.

The trenching method was applied to divide *R*_S_ into *R*_H_ and *R*_A_, the litter removal was used to calculate the respiration derived from aboveground litter decomposition. We calculate the soil respiration fractions from each source as follows:Heterotrophic respiration (*R*_H_) = NR plots

$$Autotrophic\;respiration\;(R_{A})=Control\;plots–NR\;plots$$

$$Litter\;respiration\;(R_{L})=Control\;plots–NL\;plots$$

### Soil respiration measurements

In each treatment plot, one polyvinyl chloride collar (PVC) (diameter of 20 cm and height of 8 cm) was permanently installed 2–3 cm deep into soils to measure soil respiration. All vegetation inside the collar had removed artificially before monitored soil respiration. To minimize the response caused by transient decomposition of dead roots, the initial measurements of soil respiration was carried out 3 months after trenching treatment was conducted. From November 2015 to October 2017, soil respiration was measured every month using an automated soil respiration system (Li-8100, Li-Cor Inc., Lincoln, NE, USA). Soil respiration was measured between 9.00 a.m to 12:00 a.m (Beijing time). At the same time, soil temperature (°C) and moisture (v/v) at the depth of 5 cm was synchronously measured by the probes connected to Li-8100 system.

### Microclimate and litterfall measurements

Rainfall, air temperature (T_air_) and soil temperature (T_soil_) were constantly measured using HOBO Micro Station Data Loggers (Onset Computer Corporation, Bourne, Massachusetts, USA) located adjacent to the experimental area. Forest aboveground litter was collected using six circular litter traps in each plot. The litter traps were funnel shaped with the diameter was 1 m and the collection area was 0.785 m^2^. During the monitoring period, each trap was collected monthly, then collected litter was oven-dried at 65°C to a constant mass for 48 h, and weighed.

### Statistical analyses

Based on the measured data, an exponential model was performed to describe the relationship between soil respiration fractions and soil temperature [18]:

$$R_{S}\;\left(\mathrm{or}\;R_{A},R_{H},\;R_{L}\right)=a\;\times e^{\beta \times T}$$

Where *R* was soil respiration rate (μmol m^-2^ s^-1^), *T* was soil temperature (°C), coefficient α is the intercept of soil respiration when temperature is zero, and coefficient β represents the temperature sensitivity of soil respiration. Based on the continuous soil temperature at the 5 cm depth, monthly and annual soil CO_2_ fluxes of each fraction was estimated by integrating CO_2_ fluxes for the period from November 2015 to October 2017 using the observed specific response equation between soil respiration fraction and soil temperature. Monthly and annual soil CO_2_ fluxes of each fraction was estimated by interpolating measured soil respiration between sampling dates for every day of the year and then computing the sum to obtain the annual or winter values [19].

Structural equation models (SEM) were used to assess the holistic effect of measured variables on monthly fluxes of each respiration component. In the model, monthly soil respiration emission of each component was the response variable. Monthly mean air and soil temperature, monthly precipitation (MP), litterfall production, soil moisture were variables. The normality of data distribution was examined for heteroscedasticity, and all bivariate relationships were checked for signs of nonlinearities before the SEM analysis. These analyses were performed by using the maximum-likelihood estimation. Model fit was considered good when the χ^2^ test was low (< 2) and its associated *p*-value was high (> 0.05). The Bentler’s comparative fit index (CFI) and Bentler-Bonett normed fit index (NFI) were used to evaluate the adequacy of fit. For each set of analysis, *R*^2^ values were obtained for each dependent matrix, representing the proportion of total variance explained by the model. All statistical analyses were carried out in SPSS version 20.0 for Window (SPSS Inc, Chicago, Illinois, USA). Graphs were generated using SigmaPlot 12.5 software (Systat Software, Inc., San Jose, CA, USA). Significance was determined at *α* = 0.05.

## Results

### Microclimate and litterfall

**Fig 1:**
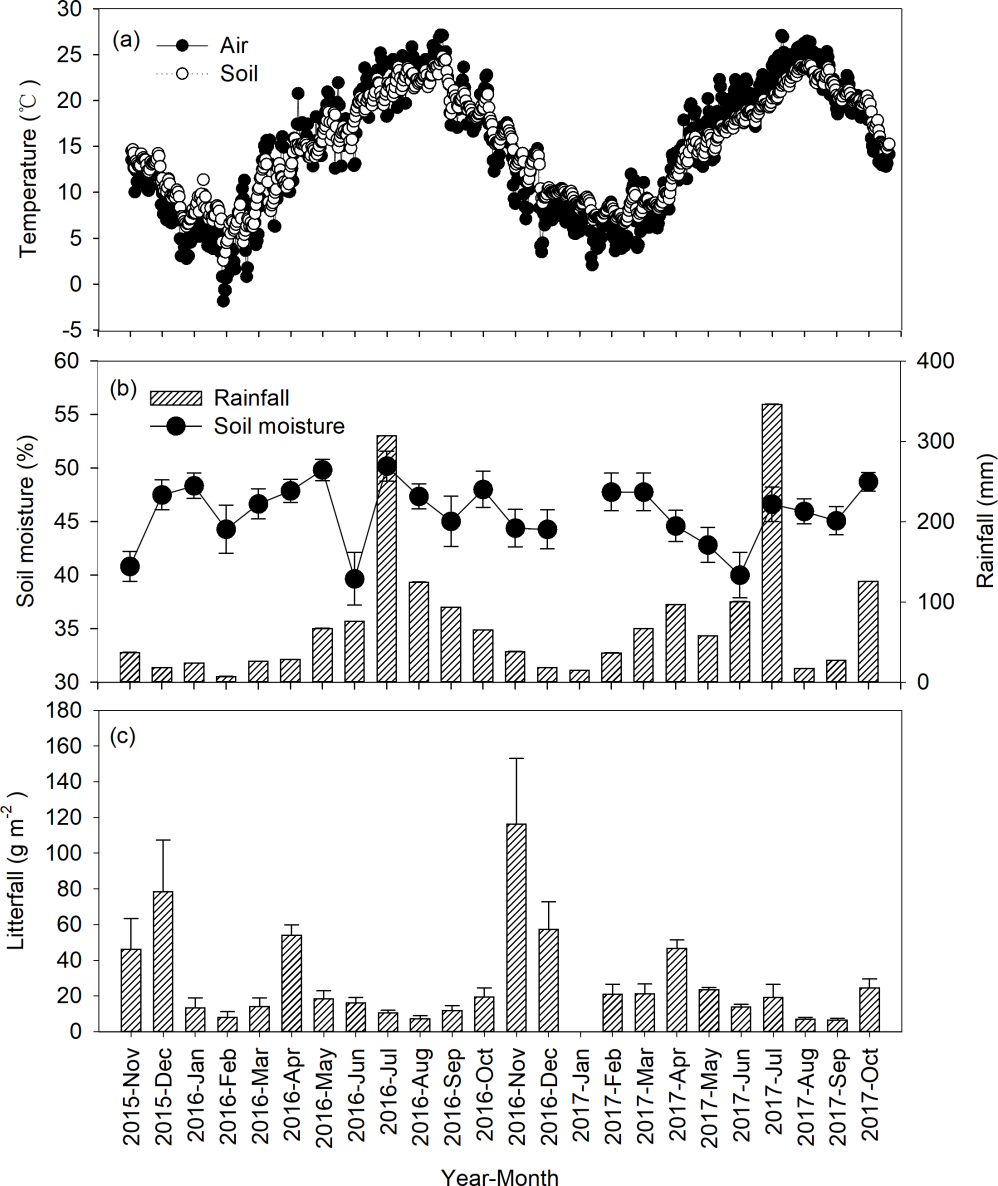
Seasonal variations of air and soil temperatures (a), precipitation and soil moisture (b), and litterfall (c) in the Michelia wilsonii plantation of southwestern China.

Both air and soil temperature showed a clear seasonal dynamic (Fig 1A). Average air temperatures was 14.6°C and 14.9°C, respectively, in 2016 and in 2017; likewise, mean soil temperatures was 15.1°C in 2016 and 17.9°C in 2017. Rainfall was 872.8 mm and 944.8 mm, respectively, in 2016 and 2017. Similar to air temperature, it was higher in summers but lower in winters (Fig 1B). However, there was no obvious seasonal variation in soil moisture and a minimum was observed in June of both years (Fig 1B). Annual aboveground litterfall was 312.0 g m^-2^ a^-1^ in 2016 and 352.2 g m^-2^ a^-1^ in 2017 (Fig 1C). In general, the amount of litterfall was significantly greater in the winter (November and December) as compared to other seasons (Fig 1C).

### Soil respiration components

**Fig 2:**
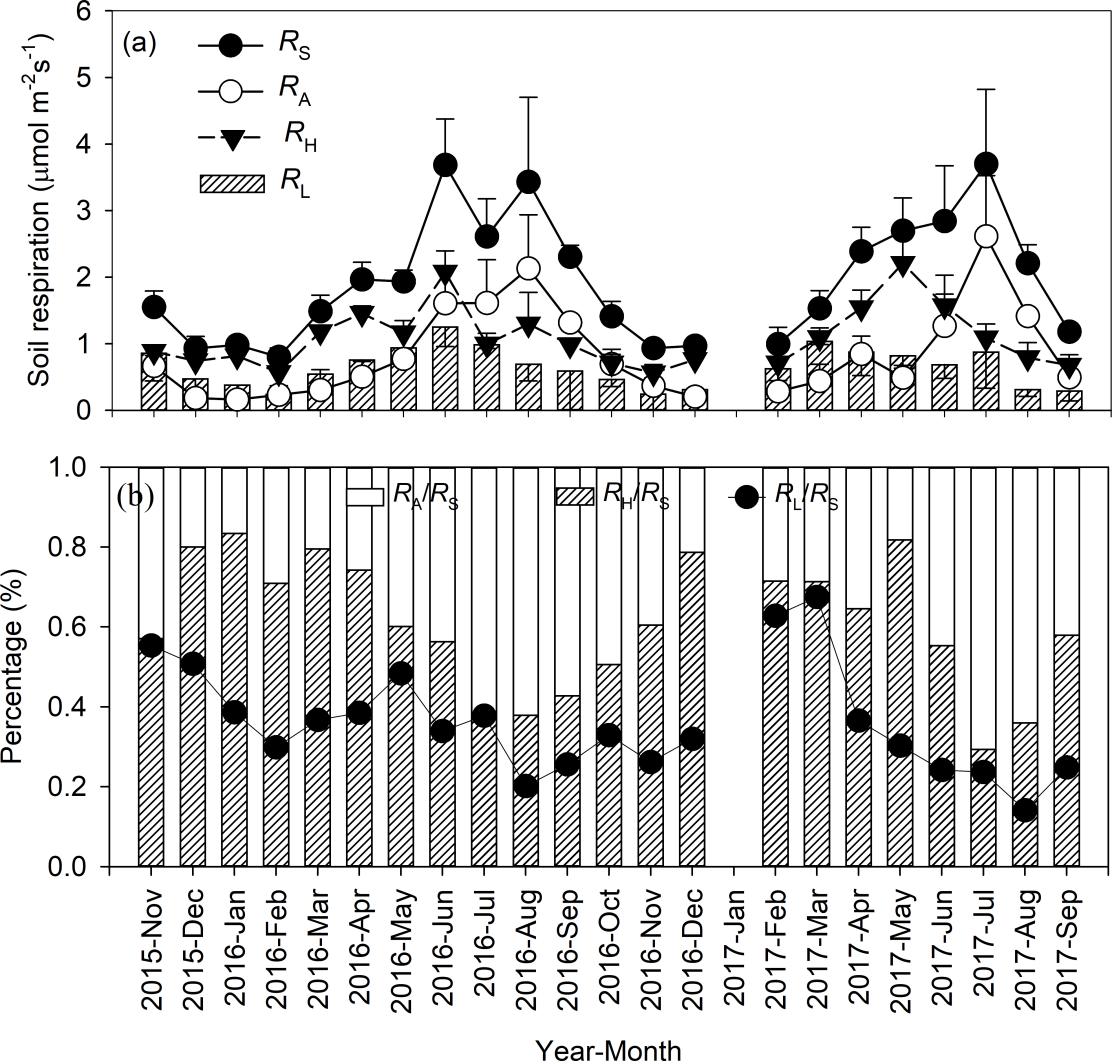
Seasonal variations of components of soil respiration rates (a) and its percentage (b) in the *Michelia wilsonii* plantation of southwestern China.

The rates of all soil respiration components had obvious seasonality, with the maximum in summer and the minimum in winter (Fig 2A). The mean annual rates of *R*_S_, *R*_A_ and *R*_H_ was 1.94 μmol·m^-2^·s^-1^, 0.85 μmol·m^-2^·s^-1^ and 1.09 μmol·m^-2^·s^-1^, respectively, throughout the experimental period (Table 1). In general, *R*_H_ was higher than *R*_A_ over the year. However, the opposite tendency was observed from June to August.

**Table 1:** Mean reparation rate and total CO_2_ emission of each component in 2016 (from November 2015 to October 2016) and 2017 (from November 2016 to October 2017) in the *Michelia wilsonii* plantation of southwestern China.

	Year	*R*_S_	*R*_A_	*R*_H_	*R*_L_
Mean respiration rate (μmol m^-1^ s^-1^)	2016	1.93	0.85	1.08	0.68
	2017	1.95	0.84	1.10	0.61
	Average	1.94	0.85	1.09	0.65
Annual CO_2_ emission (Mg hm^-2^ a^-1^)	2016	7.90	3.32	4.58	2.62
	2017	7.95	3.19	4.76	2.59
	Average	7.93	3.26	4.67	2.61

**Fig 3:**
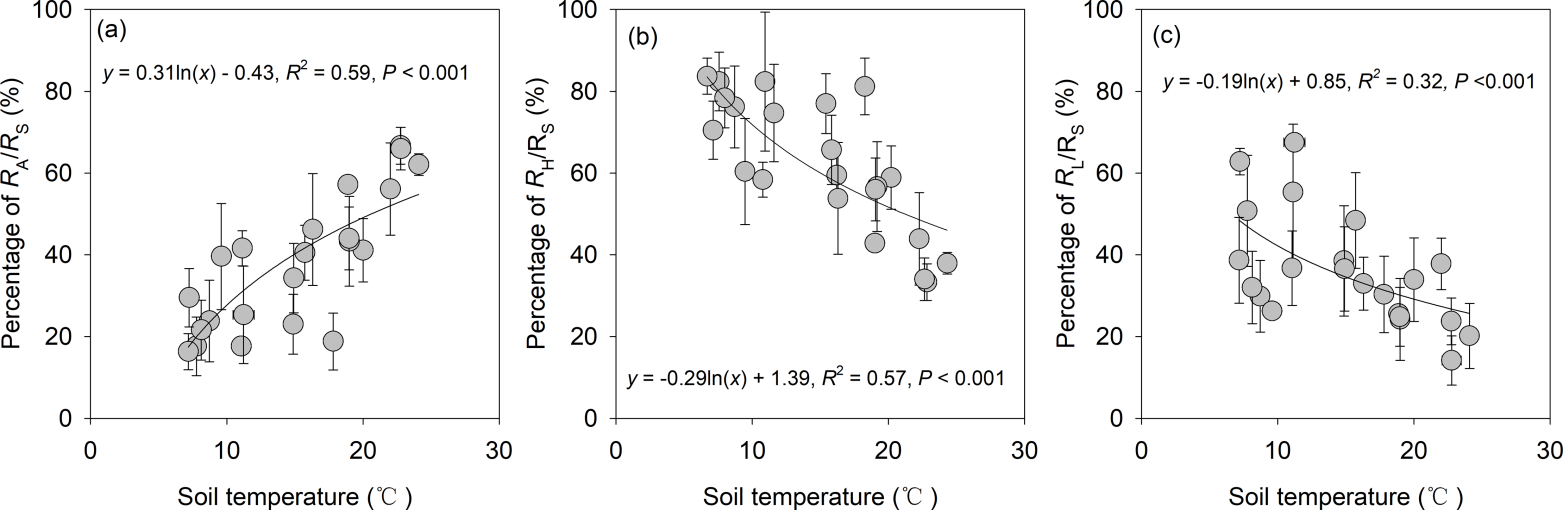
Relationships of *R*_A_/*R*_S_, *R*_H_/*R*_S_ and *R*_L_/*R*_S_ against soil temperature in the *Michelia wilsonii* plantation of southwestern China.

During the two years, the proportion of *R*_A_ to *R*_S_ (*R*_A_/*R*_S_) ranged from the maximum 70.6% in summer to the minimum 16.6% in winter, whereas the proportion of *R*_H_ to *R*_S_ (*R*_H_/*R*_S_) showed the opposite pattern (Fig 2B). The proportion of *R*_L_ to *R*_S_ (*R*_L_/*R*_S_) varied from 14.1% to 67.5%. Annual CO_2_ emission derived from R_S_, *R*_A_, *R*_H_ and *R*_L_ was 7.93 Mg C ha^-1^ a^-1^, 3.26 Mg C ha^-1^ a^-1^, 4.67 Mg C ha^-1^ a^-1^ and 2.61 Mg C ha^-1^ a^-1^, respectively (Table 1). There was a positive logarithmic relationship between *R*_A_/*R*_S_ and soil temperature (Fig 3A, *P* < 0.001). Conversely, both *R*_H_/*R*_S_ and *R*_L_/*R*_S_ decreased with increasing soil temperature (Fig 3B and 3C, *P* < 0.01).

### Correlations between environmental variables and soil respiration components

**Fig 4:**
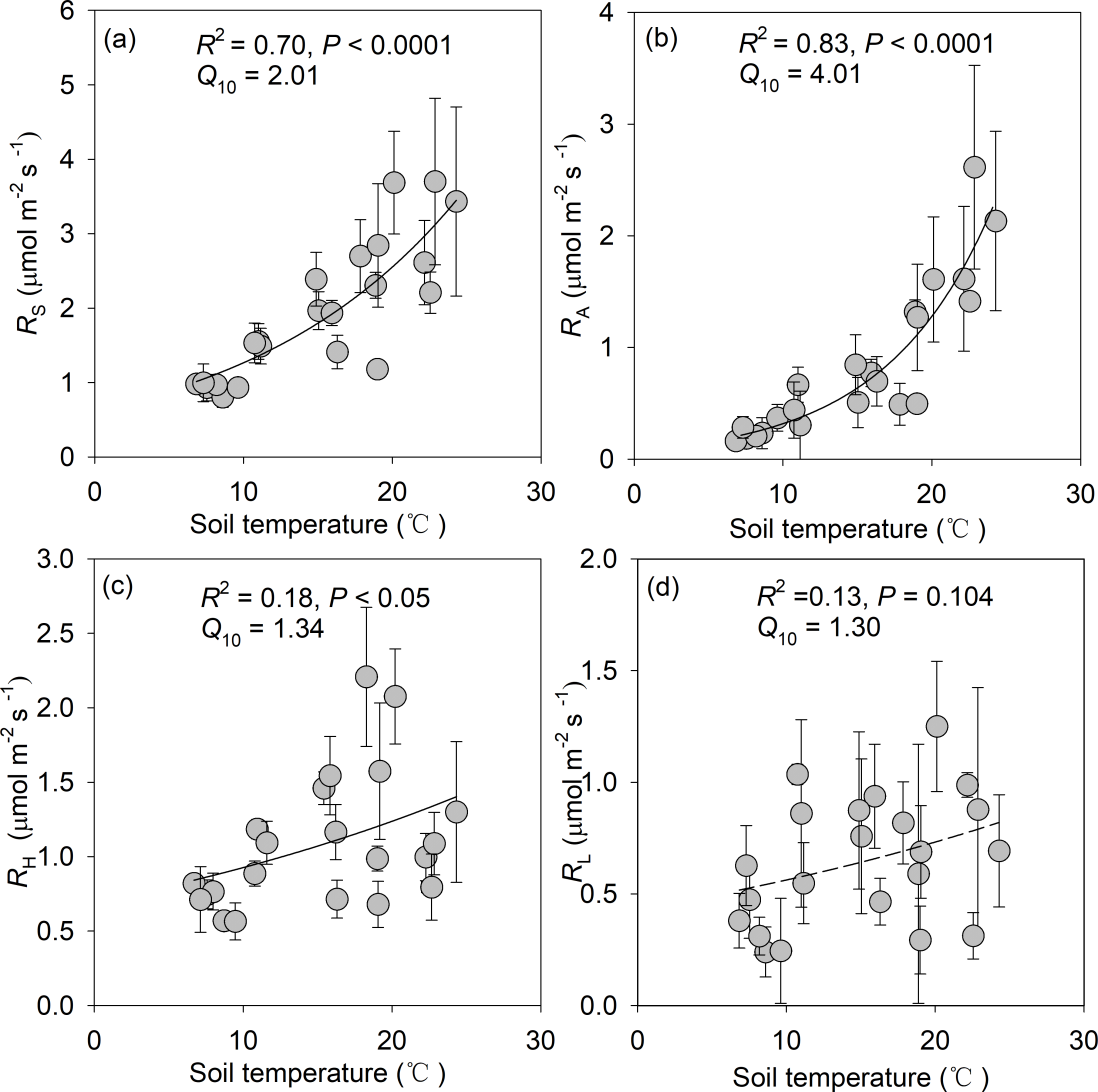
Relationships of R_S_, R_A_, R_H_ and R_L_ against soil temperature in the *Michelia wilsonii* plantation of southwestern China.

*R*_L_, *R*_S_, *R*_A_ and *R*_H_ rates all exhibited an exponential correlation with soil temperature (Fig 4). Correspondingly, the *Q*_10_ values of *R*_S_, *R*_A_ and *R*_H_ were 2.01, 4.01 and 1.34, respectively (Fig 4A–4C). Moreover, the *Q*_10_ value of *R*_A_ was higher than those of *R*_H_ and *R*_L_.

**Fig 5:**
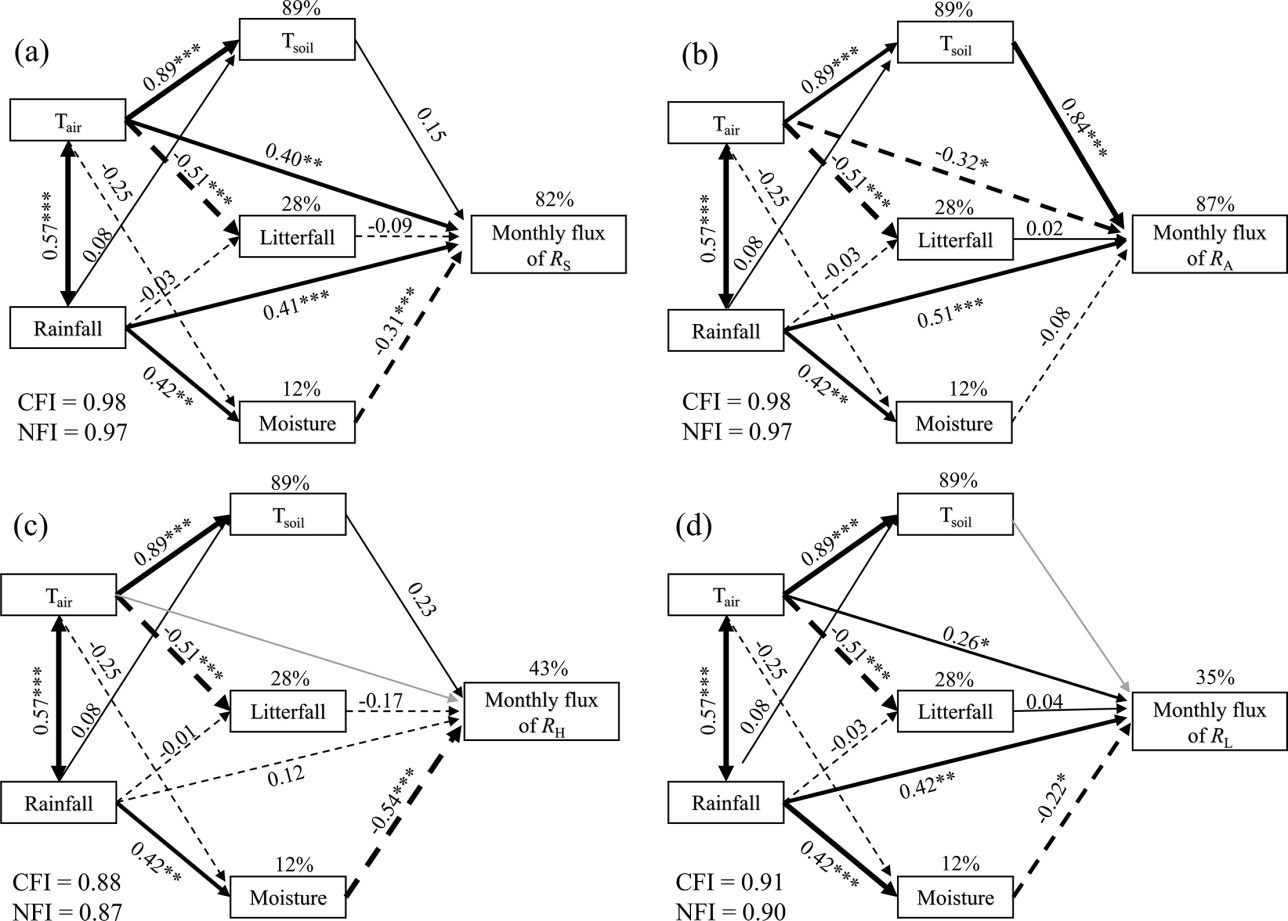
A path analysis model of the relationships among the monthly flux of soil respiration fractions, and environmental variables and litterfall in the *Michelia wilsonii* plantation of southwestern China. T_air_: monthly mean air temperature, T_soil_: monthly mean soil temperature, Rainfall: monthly rainfall amount, Litterfall: monthly aboveground litter production, Moisture: soil moisture. Solid lines are shown positive correlations path, dashed lines are shown negative paths, and gray lines mean removal paths. Standardized coefficients are listed on each path. “*” mean significant difference at 0.05 level, “**” mean significant difference at 0.01 level. “***” mean significant difference at 0.001 level.

Path analysis showed that measured variables together explained the variation of 82.0%, 86.8%, 43.0% and 34.6%, respectively, in monthly flux of *R*_S_, *R*_A_, *R*_H_ and *R*_L_ (Fig 5). Total, direct and indirect effects of the environmental variations were summarized in Fig 5. T_air_ and rainfall were strongly associated with monthly flux of *R*_S_ and *R*_L_ (Fig 5A and 5D). Moreover, T_air_ had an indirect effect on monthly flux of *R*_S_ (0.26), *R*_A_ (0.77) and *R*_H_ (0.41) via T_soil_, litterfall and moisture (Fig 5A–5C). Soil moisture had a significant negative effect on *R*_S_ (-0.31), *R*_H_ (-0.54) and *R*_L_ (-0.22) (Fig 5A, 5C and 5D).

## Discussion

### Annual C release and variations through soil respiration components

Previous synthesis showed that annual mean total CO_2_ efflux was 3.22 Mg C ha^-1^ a^-1^, 6.62 Mg C ha^-1^ a^-1^ and 10.92 Mg C ha^-1^ a^-1^, respectively, for boreal, temperate and tropical forests [2]. The mean annual soil CO_2_ effluex of Chinese subtropical forests is 10.64 Mg C ha^-1^ a^-1^, which is comparable to the reported value from global tropical forests [2]. In the current subtropical forest stand, annual *R*s emission (7.93 Mg C ha^-1^ a^-1^) is similar to the value reported in the tropical and subtropical forests (3.45–15.2 Mg C ha^-1^ a^-1^) [20]. Annual *R*s efflux in *M*. *wilsonii* forest is higher than those of *Cunninghamia lanceolata* plantation in Hunan (4.55 Mg C ha^-1^ a^-1^) and Fujian (4.54 Mg C ha^-1^ a^-1^) of eastern subtropical China [21, 22], and is comparable to those of *Castanopsis kawakamii* forest (9.34 Mg C ha^-1^ a^-1^) and *Pinus massoniana* forest (9.57 Mg C ha^-1^ a^-1^) [23], but is lower than those of *Mytilaria laosensis* (10.68 Mg C ha^-1^ a^-1^), and *Castanopsis carlesii* (11.18–12.31 Mg C ha^-1^ a^-1^) and *Cunninghamia lanceolata* forests (11.99 Mg C ha^-1^ a^-1^) in subtropical China [9, 22, 24, 25]. These differences may be attributed to climates, vegetation types or substrate quality. Previous studies suggested that mean annual temperature (MAT) and mean annual precipitation (MAP) were positively correlated with *R*s [4]. Additionally, there is also a positive relationship (*R*^2^ = 0.35, *P* = 0.02) between annual respiration flux and MAT in Chinese subtropical forest ecosystems, expect for the Mt. Ailao forest site (S1 Fig; S1 Table). Similarly, annual respiration flux is significantly positively correlated with MAP (*R*^2^ = 0.31, *P* = 0.03) in subtropical China. In our site, both MAT (15.2°C) and MAP (1 243 mm) were lower than those reported in Fujian (MAT 20.1°C and MAP 1 670 mm) [24] and in Jiangxi (MAT 17.9°C and MAP 1 469 mm) [16], respectively. As a result, lower MAT and MAP may, to some extent, account for our smaller annual *R*s flux as compared to other subtropical forests with higher MAT and MAP.

### Contribution of components to total soil respiration

The relative contributions of *R*_A_ and *R*_H_ to total *R*_S_ varied with forest types, climate and methods [26–29]. *R*_A_ contributed 14%-73% to *R*_S_ among global forest ecosystems [27]. Likewise, The *R*_H_/*R*_S_ varied from 10% to 94% among global forest ecosystems [4]. A global synthesis of forest soil respiration showed that the contribution of *R*_A_ to total *R*_S_ is higher in deciduous broadleaf forest than in evergreen broadleaf forest and evergreen needle leaf forest [4]. For example in a temperate deciduous forest in northern China, the higher *R*_A_/*R*_S_ (ranged from 61.7%–77%) is closely related to the higher root biomass associated with belowground carbon metabolisms [30]. In this case, mean *R*_A_*/R*_S_ was 41.1%, which was higher than the mean value (30%) of global forest ecosystems [2]. Similarly, the mean *R*_A_/*R*_S_ value (41.1%) estimated in this evergreen broad-leaved forest is comparable to the results observed in a monsoon evergreen broad-leaved forest (*tree species name*) (44.52%) in southern China [31], but is lower than the findings investigated in an evergreen needle forest (*Pinus massoniana*) (55–63%) or in a deciduous broadleaf forest (*tree species name*) (54–59%) [32]. As a consequence, the contribution of *R*_A_ to *R*_S_ might, to large extent, be dependent on forest functional type that determines the belowground root growth and activities [11, 30–32].

Additionally, in this study, the relative contribution of *R*_A_ and *R*_H_ to *R*_S_ largely varied with season. Over the growing seasons (from July to August) of two years, *R*_A_ contributed more to *R*_S_ as compared to *R*_H_. This is due to the fact that *R*_A_ and *R*_H_ are dominated by different mechanisms [10]. *R*_A_ is closely linked to root activity and photosynthesis, while *R*_H_ is the respiratory product of soil organic matter decomposition that mainly controlled by substrate and temperature [26]. As a result, there is a significant increase in *R*_A_ during the growing seasons in *M*. *wilsonii* forest as a result of fast root growth and large root secretions. In addition, *R*_H_/*R*_A_ increased with soil temperature but *R*_A_/*R*_A_ decreased with soil temperature. *R*_A_ was more sensitive to temperature relative to *R*_H_. Such results also can, to some extent, account for the higher *R*_H_/*R*_A_ in the growing season noted in this study.

Litter respiration is an important source of CO_2_ emission. In our study, annual CO_2_ emission from litter layer was 2.61 Mg C ha^-1^ a^-1^, which value is higher than the results observed in *Cunninghamia lanceolata* (1.15 Mg C ha^-1^ a^-1^) and *Castanopsis kawakamii* (1.17 Mg C ha^-1^ a^-1^) forests in subtropical China [5, 22], but is lower than the values reported in a *Castanopsis carlesii* forest (4.34 Mg C ha^-1^ a^-1^) [5], indicating *R*_L_ is mainly regulated by tree-associated litter quantity and quality [8]. During the experimental period, aboveground litter accounted for 14.1%–67.5% of total *R*_S_, with a mean value of 32.9%, which is close to the results found in secondary *Castanopsis carlesii* forest (34.4%) [5]. Numerous studies have evidenced that litter manipulation could markedly change total soil respiration [5, 8, 33]. Li et al. [5] found that litter addition could enhance annual CO_2_ flux by approximately 12.5%, but decreased by 15.1% when the litter was removed.

### Temperature sensitivity of soil respiration components

Over past decades, the temperature sensitivity of *R*_S_ (hereafter referred to as apparent temperature sensitivity) has gained more attention due to its importance for climate-carbon feedback in terrestrial ecosystems. In this study, the temperature sensitivity (*Q*_10_ value) of *R*_S_ was 2.01, which is similar to the mean value (2.51) of Chinese forest ecosystems [12], but is lower than the mean value (3.4) estimated for global forest ecosystems [4]. Different components of *R*_S_ may response to soil temperature differently [34]. For an example, the *Q*_10_ value for *R*_A_ and *R*_H_ was 3.74 and 1.92 in a *Castanopsis carlesii* plantation, respectively [24]. A synthesis also indicated that the *Q*_10_ value of *R*_A_ is significantly higher than that of *R*_H_ in global forest ecosystems [4]. Similarly, the *Q*_10_ value of *R*_A_ and *R*_H_ was 4.01 and 1.34, respectively in the present study, implying that *R*_A_ is more temperature-dependent as compared to *R*_H_. The C release derived from litter layer is easier to be affected precipitation as compared to *R*_A_ and *R*_H_. There was a negative relationship between *R*_L_ and soil moisture in this study. Thus, the temperature dependence of *R*_L_ could, to large extent, be offset and complicated by negative effect of soil moisture. Therefore, no significant correlation was detected between *R*_L_ and soil temperature in the present study. A significant positive linear correlation was found between *R*_A_/*R*_H_ and *Q*_10_ of *R*_A_ in Chinese subtropical forests (*R*^2^ = 0.72, *P* < 0.01, S2 Fig). Such result showed that a greater contribution of *R*_A_ to *R*_S_ may produce a higher *Q*_10_ of *R*_A_, suggesting that root activities might more sensitive to future warming [4].

### Effects of environmental factors on soil respiration components

*R*_A_ is mainly influenced by tree species and root activity [14]. However, both *R*_H_ and *R*_L_ are mainly influenced by soil temperature and substrate properties. The path analysis indicated that measured variables together explained 82% and 87% variations monthly fluxes of *R*_S_ and *R*_A,_ respectively (Fig 5A and 5B). Soil temperature is a key factor regulating the variations of two components. Contrastingly, all factors together only accounted for 43% variations in monthly flux of *R*_H_. This is attributed to the fact that season-associated changes, such as moisture or microbes, counteract the dominant effect of temperature on *R*_H_. Similar phenomena have recently been observed in grassland ecosystem [35].

Similar to *R*_H_, litter layer is stored on the surface of the forest ground. The CO_2_ emission from litter decomposition is easy to be affected directly and/or indirectly by multiple factors, including temperature, moisture, microorganisms. Path analysis showed that all factors together only explained 35% variation in monthly flux of *R*_L_. Sufficient soil moisture associated with frequent rainfall in the study area throughout the year may overshadow temperature effect [36]. A recent study also suggested that soil temperature had no significant impacts on *R*_L_ in a subtropical mixed forest [37].

Trees species-induced variation in the quantity and quality of litterfall might impact the relationship between *R*_L_ and litterfall [17]. For an instance, there was a significant relationship between *R*_L_ and litter mass of the current month in *Cunninghamia lanceolata* forest. However, *R*_L_ was significantly related to the litterfall of two months ago in *Castanopsis carlesii* forest [5]. In our site, *M*. *wilsonii* is a broad-leaved evergreen tree species, whose litterfall had a peak in winter and a sub-peak in spring. Several results have suggested a potentially lagged effect of litterfall on *R*_L_ in subtropical forests [5, 17]. Similar finding was observed in our study. There was a significant correlation between *R*_L_ rate (4 months behind) and monthly litterfall in the *M*. *wilsonii* forest (*R*^2^ = 0.46, *P* = 0.04).

## Conclusions

We separated *R*_S_ into different components (*R*_A_, *R*_H_ and *R*_L_) using trenching and litter removal techniques in a subtropical *M*. *wilsonii* forest of southwestern China. The contribution of each component to total *R*_S_ varied with seasons. *R*_A_/*R*_S_ increased with increasing soil temperature, whilst both *R*_H_/*R*_S_ and *R*_L_/*R*_S_ declined with increasing soil temperature. Path analysis showed that monthly fluxes of each component were dominated by different factors. T_soil_ and other factors can well-predict the seasonal dynamics of *R*_S_ and *R*_A_. Measured variables did not show a good correlation with *R*_H_ and *R*_L_. The results noted in this study highlight the important implication of rainfall and root phenology for soil respiration in subtropical forests in this specific region.

## Supporting information

S1 Fig ***R***_**S**_ **responded non-linearly to MAT (a), but responded linearly to MAP (b) in subtropical forests of China.** Open circle represents outlier result and was obtained from the Mt. Ailao subtropical forest data, which was not included in the regression.
(DOCX)

S2 Fig *Q_10_* of *R*_A_ was positively correlated with *R*_A_/R_S_. (DOCX)

S1 Table Values of forest CO_2_ efflux from partial Chinese subtropical forests in literature. (DOCX)
