# Supplementary material for: Soil respiration in a subtropical forest of southwestern China: Components, patterns and controls
Source: PLoS One. 2018 Sep 27;13(9):e0204341. doi: 10.1371/journal.pone.0204341 (PMC6160061; doi:10.1371/journal.pone.0204341)
Supplement: S1 Fig — RS responded non-linearly to MAT (a), but responded linearly to MAP (b) in subtropical forests of China. Open circle represents outlier result and was obtained from the Mt. Ailao subtropical forest data, which was not included in the regression. (DOCX) [file pone.0204341.s001.docx]

**S1 Figure. *R*_S_ responded non-linearly to MAT (a), but responded linearly to MAP (b) in subtropical forests of China (details of the data are shown in S1 Table).** Open circle represents outlier result and was obtained from the Mt. Ailao subtropical forest data, which was not included in the regression.
