# Supplementary material for: Soil respiration in a subtropical forest of southwestern China: Components, patterns and controls
Source: PLoS One. 2018 Sep 27;13(9):e0204341. doi: 10.1371/journal.pone.0204341 (PMC6160061; doi:10.1371/journal.pone.0204341)
Supplement: S2 Fig — (DOCX) [file pone.0204341.s002.docx]

**S2 Figure. *Q*_10_ of *R*_A_ was positively correlated with R_A_/R_S_ (details of the data are shown in S1 Table).**
