# Supplementary material for: Soil respiration in a subtropical forest of southwestern China: Components, patterns and controls
Source: PLoS One. 2018 Sep 27;13(9):e0204341. doi: 10.1371/journal.pone.0204341 (PMC6160061; doi:10.1371/journal.pone.0204341)
Supplement: S1 Table — (DOCX) [file pone.0204341.s003.docx]

**S1 Table.** **Values of forest CO_2_ efflux from partial Chinese subtropical forests in literature**

| Vegetation types | Location | latitude | Longitude | MAT | MAP | R_S_ efflux  (C ha^-1^ a^-1^) | R_S_ rate  (μmol m^-2^ s^-1^) | R_A_  rate  (μmol m^-2^ s^-1^) | R_H_ rate  (μmol m^-2^ s^-1^) | R_L_ rate  (μmol m^-2^ s^-1^) | R_A_/R_S_  (%) | R_H_/R_S_  (%) | R_L_/R_S_  (%) | R_S_-*Q*_10_ | R_A_-*Q*_10_ | R_H_-*Q*_10_ | R_L_-*Q*_10_ | Reference |
| --- | --- | --- | --- | --- | --- | --- | --- | --- | --- | --- | --- | --- | --- | --- | --- | --- | --- | --- |
| Pinus massoniana | Guangdong | 23°09’ | 112°30’ | 21.4 | 1927 | 16.92 | 4.16 | 1.65 | 2.51 | / | 39.48 | 60.52 | / | 2.36 | 3.16 | 2.16 | / | Han 2011 |
| Coniferous and broad-leaved mixed | Guangdong | 23°09’ | 112°30’ | 21.4 | 1927 | 13.46 | 3.89 | 1.33 | 2.57 | / | 33.29 | 66.71 | / | 2.38 | 3.56 | 2.18 | / | Han 2011 |
| Moonsoon evergreen broad-leaved | Guangdong | 23°09’ | 112°30’ | 21.4 | 1927 | 14.15 | 4.90 | 2.21 | 2.69 | / | 44.25 | 55.75 | / | 2.95 | 4.02 | 2.25 | / | Han 2011 |
| Natural forest | Yunnan | 24°32’ | 101°01’ | 11.3 | 1840 | 12.48 | / | / | / | / | 16.01 | / | 18.73 | 3.05 | 1.65 | / | 7.22 | Tan 2013 |
| Castanopsis carlesii | Fujian | 26°11’ | 117°28' | 20.1 | 1670 | 12.31 | 3.48 | 1.22 | 2.31 | / | 32.50 | 67.50 | / | 2.41 | 1.92 | 3.74 | / | Wu 2014 |
| Cunninghamia lanceolata | Fujian | 26°12’ | 117°29' | 20.1 | 1670 | 9.06 | 2.45 | 0.92 | 1.72 | / | 24.10 | 75.90 | / | 2.12 | 1.82 | 3.00 | / | Wu 2014 |
| Castanopsis kawakamii | Fujian | 26°11’ | 117°26’ | 19.1 | 1749 | 13.74 | 3.71 | 1.70 | 0.84 | / | 47.80 | 52.20 | 27.82 | / | / | / | / | Yang 2007 |
| Castanopsis kawakamii | Fujian | 26°11’ | 117°27’ | 19.1 | 1749 | 9.44 | 2.46 | 1.06 | 1.45 | / | 42.10 | 57.90 | 28.68 | / | / | / | / | Yang 2007 |
| Chinese fir | Fujian | 26°11’ | 117°27’ | 19.1 | 1749 | 4.54 | 1.16 | 0.46 | 0.68 | / | 40.30 | 59.70 | 27.62 | / | / | / | / | Yang 2007 |
| Castanopsis carlesii | Fujian | 26°19’ | 117°36’ | 19.1 | 1749 | 12.64 | 3.41 | / | / | 1.17 | / | / | 34.40 | 1.75 | / | / | 3.44 | Li 2016 |
| Cunninghamia lanceolata | Fujian | 26°19’ | 117°36’ | 19.1 | 1749 | 7.67 | 2.07 | / | / | 0.31 | / | / | 15.10 | 1.83 | / | / | 1.51 | Li 2016 |
| Pinus massoniana Lamb | Jiangxi | 26°44’ | 115°03’ | 17.9 | 1469 | 9.75 | / | 1.08 | 1.74 | / | 55.10 | 44.90 | / | / | / | / | / | Wang 2015a |
| Pinus elliottii Engelm | Jiangxi | 26°44’ | 115°03’ | 17.9 | 1469 | 7.89 | / | 0.52 | 1.71 | / | 31.60 | 68.40 | / | / | / | / | / | Wang 2015a |
| Cunninghamia lanceolata | Fujian | 26°48’ | 117°58’ | 17.1 | 1422 | / | 2.03 | 0.44 | 1.49 | 0.29 | / | / | / | / | / | / | / | Huang 2014 |
| Mytilaria laosensis | Fujian | 26°48’ | 117°58’ | 17.1 | 1422 | / | 2.70 | 0.64 | 1.68 | 0.51 | / | / | / | / | / | / | / | Huang 2014 |
| Cunninghamia lanceolata | Hunan | 28°06’ | 113°01’ | 17.2 | 1422 | 4.55 | 1.22 | 0.34 | 0.88 | / | 33.00 | 67.00 | / | / | / | / | / | Tian 2011 |
| Mixed forest | Chongqing | 29°41’ | 106°17’ | 13.6 | 1612 | / | 2.72 | 0.74 | 1.98 | / | 26.75 | 73.25 | / | / | / | / | / | Yu 2015 |
| Evergreen Broadleaf Forest | Sichuan | 30°44’ | 103°27’ | 15.2 | 1243 | 10.83 | 3.89 | 0.60 | 3.25 | / | 51.87 | 48.13 | 17.29 | 3.46 | / | 6.26 | / | Wang 2015b |
| Michelia wilsonii | Sichuan | 30°59’ | 103°37’ | 14.7 | 1423 | 7.93 | 1.94 | 0.79 | 1.03 | 0.66 | 44.25 | 56.15 | 35.10 | 2.01 | 4.01 | 1.34 | 1.30 | this study |
| Secondary forest | Jiangsu | 32°11’ | 118°42’ | 15.5 | 1020 | / | 3.42 | 1.06 | 2.36 | / | 31.00 | 69.00 | / | 1.97 | 3.31 | 1.76 | / | Shen 2011 |

Note: MAT: mean annual temperature (℃); MAP: mean annual precipitation (mm); “/” means no data or not mentioned.

1. Han TF, Zhou GY, Li YL, Liu JX, Zhang DQ. Partitioning soil respiration in lower subtropical forests at different successional stages in southern China. Chin J Plant Ecol. 2011; 35: 946-954.
2. Huang ZQ, Yu ZP, Wang MH. Environmental controls and the influence of tree species on temporal variation in soil respiration in subtropical China. Plant Soil. 2014; 382: 75-87.
3. Li XJ, Liu XF, Xiong DC, Lin WS, Lin TW, Shi YW, et al. Impact of litterfall addition and exclusion on soil respiration in Cunninghamia lanceolata plantation and secondary Castanopsis carlesii forest in mid-subtropical China. Chin J Plant Ecol. 2016; 40: 447-457.
4. Shen, XS, Chen ST, Hu ZH, Shi YS, Zhang Y. Investigation of hetertrophic and autotrophic components of soil respiraiton in a secondary forest in subtropical China. Environ Sci. 2011, 32: 3181-3187.
5. Tan ZH, Zhang YP, Liang NS, Song QH, Liu YH, You G, et al. Soil respiration in an old-growth subtropical forest: Patterns, components, and controls. J Geophys Res: Atmos. 2013; 118: 2981-2990.
6. Tian DL, Wang GJ, Peng YY, Yan WD, Fang X, Zhu F, et al. Contribution of autotrophic and heterotrophic respiration to soil CO_2_ efflux in Chinese fir plantations. Aust J Bot. 2011; 59: 26-31.
7. Wang YD, Wang HM, Xu MJ, Ma ZQ, Wang ZL. Soil organic carbon stocks and CO_2_ effluxes of native and exotic pine plantations in subtropical China. Catena. 2015a; 128: 167-173.
8. Wang JJ, Lai LM, Zhao XC; Jiang LH, Wang YJ, Zhou JH, et al. Litter priming and trenching greatly affect soil respiration in a mature subtropical evergreen broadleaf forest in Southwestern China. Contemp Probl Ecol. 2015b; 8: 486-494.
9. Wu JJ, Yang ZJ, Liu XF, Xiong DC, Lin WS, Chen CQ, et al. Analysis of soil respiration and components in Castanopsis carlesii and Cunninghamia lanceolata plantations. Chin J Plant Ecol. 2014; 38: 45-53.
10. Yang YS, Chen GS, Guo JF, Xie JS, Wang XG. Soil respiration and carbon balance in a subtropical native forest and two managed plantations. Plant Ecol. 2007; 193: 71-84.
11. Yu L, Wang YJ, Wang YQ, Sun SQ, Liu LZY. Quantifying components of soil respiration and their response to abiotic factors in two typical subtropical forest stands, southwest China. PloS One. 2015; 10, e0117490.
